# Diversity, Ecology, and Prevalence of Antimicrobials in Nature

**DOI:** 10.3389/fmicb.2019.02518

**Published:** 2019-11-14

**Authors:** Megan M. Mullis, Ian M. Rambo, Brett J. Baker, Brandi Kiel Reese

**Affiliations:** ^1^Department of Life Sciences, Texas A&M University Corpus Christi, Corpus Christi, TX, United States; ^2^Department of Marine Science, University of Texas Marine Science Institute, Port Aransas, TX, United States

**Keywords:** antimicrobial, PKS, NRPS, competition, subsurface, diversity

## Abstract

Microorganisms possess a variety of survival mechanisms, including the production of antimicrobials that function to kill and/or inhibit the growth of competing microorganisms. Studies of antimicrobial production have largely been driven by the medical community in response to the rise in antibiotic-resistant microorganisms and have involved isolated pure cultures under artificial laboratory conditions neglecting the important ecological roles of these compounds. The search for new natural products has extended to biofilms, soil, oceans, coral reefs, and shallow coastal sediments; however, the marine deep subsurface biosphere may be an untapped repository for novel antimicrobial discovery. Uniquely, prokaryotic survival in energy-limited extreme environments force microbial populations to either adapt their metabolism to outcompete or produce novel antimicrobials that inhibit competition. For example, subsurface sediments could yield novel antimicrobial genes, while at the same time answering important ecological questions about the microbial community.

## Introduction

Microbes play fundamental roles in ecosystem functioning, particularly through mediating biogeochemical cycles, yet we know very little about their interactions in nature. Microorganisms have several mechanisms for survival depending on the respective environment: (1) outcompete neighboring populations through adaptation and/or evolution; (2) work with their neighbors *via* mutualistic cooperation; and/or (3) inhibit or kill their neighbors. One of the most common mechanisms for inhibition or elimination of competition is the production of antimicrobial compounds, including antibacterials and antifungals. These compounds can be toxic to the surrounding community, providing a selective advantage for nutrients, carbon, and space (Bibb, 2005; Rigali et al., 2008). Many environments have been explored for novel antimicrobial discovery including continental soils (Wright, 1956; Gottlieb, 1976; Chander et al., 2005; Bundale et al., 2015), caves (Cheeptham and Saiz-Jimenez, 2015), desert soils (Hozzein et al., 2011; El-Deeb et al., 2013; Nithya et al., 2015; Ouchari et al., 2019), freshwater sediments and water (Cross, 1981; Cannell et al., 1988; Madhumathi et al., 2011), and marine sediments and water (Duff et al., 1966; Martins et al., 2008; Valli et al., 2012; Bose et al., 2015; Zinke et al., 2017; Quintero et al., 2018; Ser et al., 2018; Hook and Plante, 2019; Skočibušić et al., 2019). Terrestrial areas including soils, deserts, and freshwater lakes and rivers are typically easier to access relative to deep-sea environments and generate promising results in the hunt for antimicrobial activity, as natural products isolated from these environments have been used medicinally (Mcdonald et al., 1996; Haefner, 2003; Hughes et al., 2008; Dias et al., 2012; David et al., 2015). However, one of the most diverse biomes on our planet has yet to be examined for antimicrobial production: the marine deep subsurface biosphere.

This review will survey natural antimicrobials that have been isolated from Bacteria and putative antimicrobial production within different environments including continental soil, caves, freshwater, marine, and deep subsurface sediments. Key studies focusing on specific genes for antimicrobial production within these environments will be discussed. Notably absent from this review is fungal antibiotic discovery in ecosystems other than terrestrial soils, which would necessitate an entire review on its own. The overall purpose of this review is to highlight studies that have found natural antimicrobial producing genes from Bacteria and Archaea within various environments, as well as pinpoint areas that have yet to be explored.

## History of Antimicrobial Discovery

**Figure 1:**
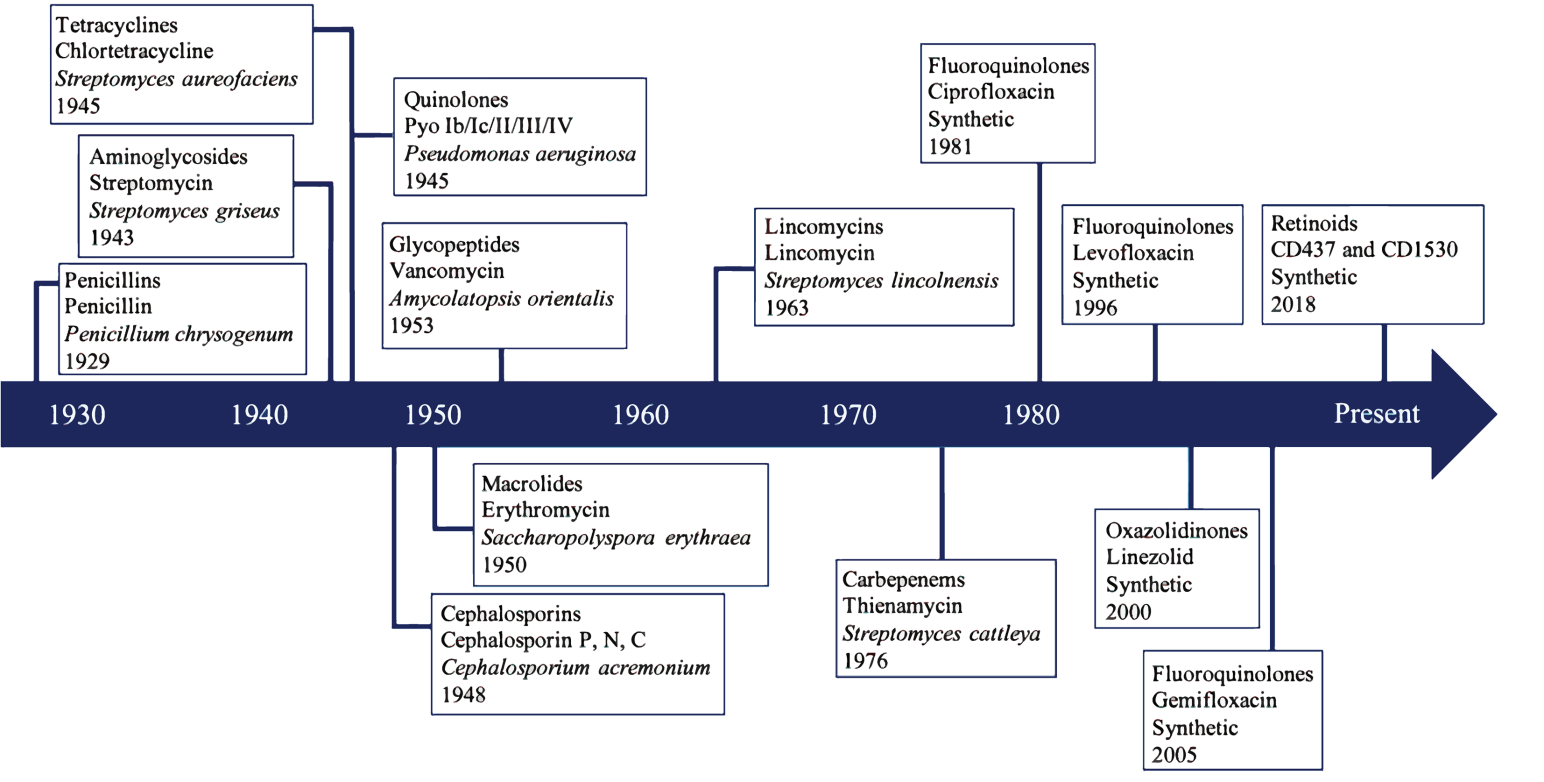
Discovery timeline of most clinically important antibiotic classes and first isolated antibiotic compound. Each box contains the class of antibiotics, the first compound isolated, the organism that naturally produced the compound, and the isolation year.

The production of an antimicrobial by a microorganism was first noted in the fungus *Penicillium chrysogenum* in 1929 by Alexander Fleming (Shama, 2009; Bush, 2010). This critical observation initiated the discovery and isolation of penicillin in the early 1940s, spurring a “Golden Age” of antimicrobial discovery from 1940 to 1980 (Figure 1), which led to a boom in the exploration, isolation, and commercialization of natural products from cultivated continental microorganisms (Mehta et al., 2006; Katz and Baltz, 2016). This Golden Age yielded natural products including biopharmaceutical primary and secondary metabolites, such as macrolides, quinolones, tetracyclines, and cephalosporins as well as chemical derivatives that combat bacteria, fungi, and eukaryotic parasites (Harvey, 2008) by inhibiting the synthesis of cell walls, proteins, DNA, or metabolites essential for cellular functions (Walsh, 2000). The initial compounds isolated from microorganisms during the Golden Age were from soils around the world including the United States, Russia, Borneo, and the Philippines (Raper et al., 1944; Hays et al., 1945; Wells, 1952; Abraham, 1962; Kaplan and Weinstein, 1968; Rubin and Tamaoki, 2000; Andersson and MacGowan, 2003; Emmerson and Jones, 2003; Grabowski, 2005; Moellering, 2006; Houbraken et al., 2011; Chander et al., 2015; Ventola, 2015; Gradnigo et al., 2016; Kim et al., 2018). After the discovery of carbapenems in 1976, antimicrobial bioprospecting from *Streptomyces cattleya* tapered off and has since been limited to artificial synthesis and rediscovery (Figure 1; Williamson et al., 1985; Drews, 2000; Aminov, 2009).

Traditionally testing for antimicrobial production relied on agar plate cultivation and identifying zones of inhibition caused by competing Bacteria (Schatz et al., 1944; Mehta et al., 2006; Lewis, 2013). Antimicrobial compounds are naturally produced as secondary metabolites from microorganisms as a defense mechanism. Microorganisms can use various methods to defend themselves from harm including producing toxins such as antimicrobial compounds that act against surrounding microbial cells. As a result, microorganisms exposed to natural products with toxic effects can develop strategies for antimicrobial resistance (Cohen, 1992; Tenover, 2006), which are observed relatively quickly in microorganisms due to horizontal gene transfer (Read and Woods, 2014). Naturally-produced antimicrobials and antimicrobial resistance have co-evolved within microbial communities by eliminating susceptible strains, leaving behind those that have resistance or become resistant (Kerr et al., 2002; Ventola, 2015). This means that the method of culturing Bacteria to identify and isolate bioactive compounds often results in the re-discovery of known compounds, which is expensive and time consuming (Machado et al., 2015). However, computational methods for assessing secondary metabolite production potential can complement, or in some cases replace, traditional exploration techniques (Culligan et al., 2014; Adu-Oppong et al., 2017).

## Polyketide Synthase and Nonribosomal Peptide Synthase Gene Structure and Function

Antimicrobial production occurs through a multitude of metabolic intermediate biosynthesis pathways, with two of the most common and extensively researched protein families involved in these processes being the polyketide synthases (PKS) and non-ribosomal peptide synthases (NRPS) (Ehrenreich et al., 2005). PKSs synthesize polyketides, a broad class of bioactive compounds defined by alternating carbonyl and methylene groups (Staunton and Weissman, 2001), while NRPSs produce peptides independent of messenger RNA and ribosomal machinery (Walsh, 2016). PKSs and NRPSs exhibit high structural and functional similarity and have been found to form hybrid genes producing antimicrobial compounds (Fisch, 2013). These genes are generally organized into operons or gene clusters generally ranging from 1 kb to greater than 10 kb in length (Medema et al., 2015) which encode large, multifunctional enzymes (200–2,000 kDa) (Ehrenreich et al., 2005). PKSs and NRPSs can synthesize other biologically active compounds such as siderophores and immunosuppressants (Cane and Walsh, 1999; Crosa and Walsh, 2002; Ehrenreich et al., 2005). These enzymes are classified according to their structure and biosynthetic function.

There are three types of naturally occurring PKS modules, aptly designated Type I, II, and III (Shen, 2003). Type I is composed of large enzymes that contain multiple functional domains with defined functions that perform a single catalytic step during biosynthesis of the antimicrobial compound. The Type II PKS complex includes several single-module proteins with separated enzymatic activities acting repetitively to produce a polyketide. Type III consists of a single active site enzyme that acts iteratively to form the final polyketide product (Austin and Noel, 2003; Weissman, 2009). Type I is most common in Bacteria, Type II is most commonly found in fungi, and Type III is most common within plants (Yu et al., 2012). Type I and II PKS genes will receive focus in this review.

There are approximately 10 domains that contribute to the PKS gene. Three essential domains are necessary for the PKS operon to function, which include β-ketosynthase (KS), acyltransferase (AT), and acyl carrier protein domains (ACP) (Ansari et al., 2004). The KS domain functions *via* the attachment of a malonyl-CoA extender unit to an acetyl-CoA starter molecule. The AT domain serves as a support to load the appropriate substrate onto the enzyme, whereas the ACP domain supervises the movement of substrates and products between the different active sites of the enzyme. Variability of antimicrobial compounds produced *via* the PKS pathway is due to insertion or deletion of optional domains, including β-ketoreductase, dehydratase, and trans-acting enoyl (Cane and Walsh, 1999). If all three of these optional domains are present, then the PKS gene is highly reducing, otherwise it is non-reducing (Ma et al., 2009; Gallo et al., 2013; Liu et al., 2015). This determination of highly-reducing versus non-reducing is due to the presence of domains that can reduce and/or dehydrate substrates to construct a specific compound (Chiang et al., 2010). PKS genes can be deemed partially-reducing if the β-ketoreductase and dehydratase domains are present and the trans-acting enoyl domain is not. There are other optional domains that are less prevalent than those described. Non-reducing PKS genes have a special domain located in at the N-terminus called the starter unit acyl carrier protein transacylase, which is responsible for selection and loading of a starter unit and the product template. The product template domain controls the folding of the polyketide backbone (Gallo et al., 2013).

The NRPS gene encodes multifunctional enzymes whose modules elongate the amino acid chain (Staunton and Weissman, 2001). Similar to the PKS gene, there are three essential domains required for the function of this gene. They include the adenylation (A), thiolation/peptidyl carrier protein (T/PCP), and condensation domains (C) (Weissman and Müller, 2008; Weissman, 2015). The A domain recognizes and activates the related amino acid or hydroxyl acid. The T/PCP domain functions as a “swinging arm” carrying a phosphopantetheinyl at a conserved serine residue and delivers substrates to the corresponding active site of the domain. The C domain catalyzes the peptide bond formation between the activated amino acid and elongation chain (Weissman, 2015). There are optional domains associated with NRPSs as well, such as the epimerization, reductase, cyclization, and oxidation domains (Gallo et al., 2013). It has been proposed that NRPSs can be sorted into three groups: linear, iterative, and non-linear (Mootz et al., 2002; Eisfeld, 2009). Linear NRPSs follow a specific template of domain organization and can be predicted (Eisfeld, 2009). Iterative NRPSs are similar to linear NRPSs but use some domains repeatedly during biosynthesis. These NRPSs are more common with fungi and are better described due to predictability (Keller et al., 2005; Medema et al., 2011; Röttig et al., 2011). Non-linear NRPSs have more complex domain interactions and the possible product created cannot be predicted (Mootz et al., 2002). The inability to predict non-linear NRPS products is due to the capability to utilize free soluble molecules during biosynthesis (Eisfeld, 2009).

Hybrid PKS-NRPSs have been found to be structurally and functionally diverse across bacteria (Wenzel and Müller, 2005a,b; Fisch, 2013). Microbial hybrid PKS-NRPSs are formed commonly in nature by adjoining the PKS modules and NRPS modules together in an ‘assembly line’ fashion (Fisch, 2013). The functionality of the hybrid depends on the domain organization within each module. The organization of the domains within each module results in a specific cascade of enzymatic reactions that give rise to diverse hybrid antimicrobials such as bleomycin (Du et al., 2000), epothilone (Tang et al., 2000), yersiniabactin (Pelludat et al., 1998), and rapamycin (Aparicio et al., 1996; Boettger and Hertweck, 2013; Masschelein et al., 2013).

**Figure 2:**
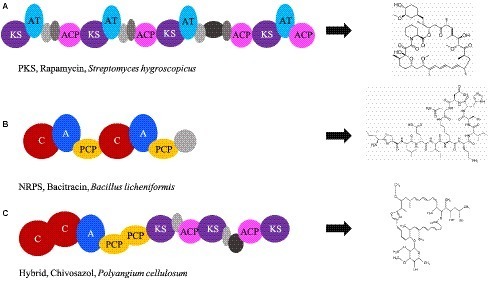
Essential and optional domains of PKS and NRPS. The order and combination in which domains appear in the genome determines which antimicrobial compound is produced. Examples include: **(A)** PKS domain order that is associated with production of rapamycin (accession number X86780.1); **(B)** NRPS domain order that is associated with production of bacitracin (accession number EF159954); **(C)** domain order of PKS and NRPS that is associated with Chivosazol (accession number DQ065771). KS, ketosynthase; AT, acyltransferase; ACP, acyl carrier protein; C, condensation; A, adenylation; PCP, peptidyl carrier protein. Gray ovals depict domains that are nonessential but add variability to the antimicrobial compound. The domains were visualized from sequences using ClusterMine360 and antiSMASH.

Both PKS and NRPS pathways are diverse due to the variable domains that can be present or absent in a specific order following the essential domains (Figure 2; Cane and Walsh, 1999). The order, presence, absence, and essential domains are critical to what antimicrobial molecule is created. Due to the large size and complexity of antimicrobial gene clusters, it is difficult to target the entire antimicrobial gene for identification and isolation (Weissman, 2015; Ziemert et al., 2016). However, the identification, isolation, and availability of essential domain sequences in public databases have aided further annotation of homologous domains of these megasynthases through bioinformatic data mining (Keller et al., 2005; Bergmann et al., 2007; Khaldi et al., 2010; Weber et al., 2015). Despite these recent advances, only a few studies exist that explore these genes in an ecological context (Helfrich et al., 2014; Tyc et al., 2017).

## Computational Approaches to Discovery

Limitations in culture-based techniques have brought computational genomic mining methods to the forefront of antimicrobial exploration (Banik and Brady, 2010; Juhas et al., 2011). Advances in DNA sequencing technologies and computational approaches have become increasingly prevalent since the early 2000s as a means of exploration and prediction of antimicrobial production potential (Van Lanen and Shen, 2006; Li and Vederas, 2009; Zerikly and Challis, 2009; Harvey et al., 2015). The study of “omics includes analysis of molecules” roles, actions, and relationships within a cell (Patti et al., 2012) to determine a microorganisms’ genetical potential and activity within its respective environment. The significantly decreased cost of DNA sequencing in the past decade has led to the creation of massive genomic databases (Shendure et al., 2017), with a recent explosion of environmental genomes reconstructed through metagenomic (community genetic potential, DNA) sequencing and binning methods that represent novel, and often unculturable, taxonomic lineages (Hug et al., 2016). Other ‘omics’ technologies are utilized in secondary metabolite prospecting, including metatranscriptomics (actively expressed genetic material, RNA), metaproteomics (entirety of proteins), and metabolomics (entirety of metabolites) (Zhang et al., 2010; Franzosa et al., 2015; Prosser, 2015; Beale et al., 2016; Manzoni et al., 2016). Several bioinformatic tools have been developed that enable the prediction and identification of secondary metabolites from environmental samples, such as Natural Product Domain Seeker (NaPDoS) (Ziemert et al., 2012), antibiotics and Secondary Metabolite Analysis Shell (antiSMASH) (Medema et al., 2011), NRPSpredictor2 (Röttig et al., 2011), and Environmental Surveyor of Natural Product Discovery (eSNaPD). The NaPDoS pipeline searches genomic or metagenomic data for the presence of the KS domain for PKSs and the C domain for NRPS (Ziemert et al., 2012). This program allows for insight into putative antimicrobial production but is limited due to analysis of only one essential domain for PKSs and NRPSs. The antiSMASH pipeline identifies known antimicrobial production loci using profile hidden Markov models and aligns them with regions of the closest relative gene cluster (Medema et al., 2011), while NRPSpredictor2 utilizes support vector machine-based learning to predict domains (Röttig et al., 2011). The eSNaPD platform can be used for discovery of novel natural products or derivatives of known antimicrobial compounds from metagenomic data (Reddy et al., 2014). While these tools are useful in searching for antimicrobial producing genes, it must be considered that data mining approaches are only as effective as the reference databases provided (Scholz et al., 2012; Kim et al., 2013; Hiraoka et al., 2016; Siegwald et al., 2017).

**Figure 3:**
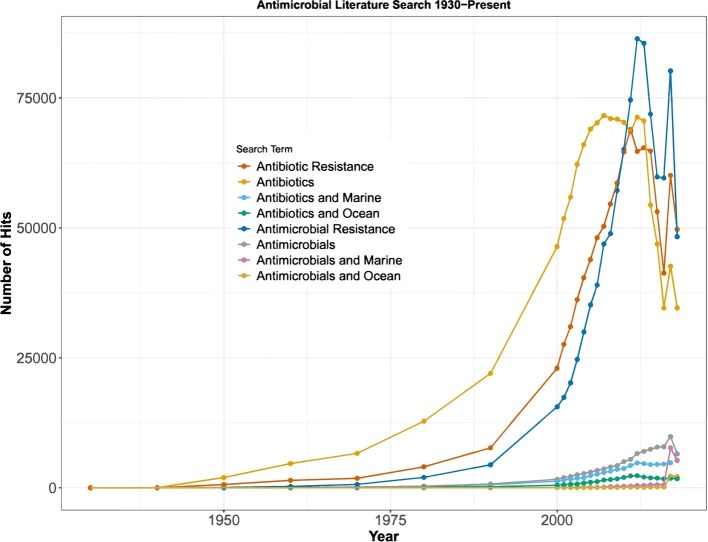
Prevalence of published peer-reviewed literature containing each search term. The earliest study to use the term “antibiotic” was published in 1930. From 1930 to 2000, “hits” were recorded in 10-year increments, and from 2000 to 2018, the number of “hits” was recorded for each year.

Databases specific to the PKS and NRPS antimicrobial genes are ClusterMine360 (Conway and Boddy, 2012) and antiSMASH (Blin et al., 2016), which include all of the essential domains needed for antimicrobial synthesis. These manually curated databases can be utilized for mining antimicrobial producing genes from publicly-accessible sequencing data, which lends itself to a unique opportunity as well as a massive challenge. To date, there are approximately 200,000 genomes, 40,000 metagenomes, and 5,000 metatranscriptomes from various environments in publicly available databases (e.g., National Center for Biotechnology Informatics). Mining these data for antimicrobial production using available bioinformatics tools may demonstrate the potential for antimicrobial production at genome-resolved or community-level scales. This has been reflected in sharp rise in the number of publications since 1990 that have included antibiotic or antimicrobial as a keyword or in the title (Figure 3).

## Antimicrobials as a Weapon or a Tool

Exploration for natural antimicrobial production has extended to a wide variety of habitats including continental soils (Gottlieb, 1976; Williams and Vickers, 1986; Laskaris et al., 2010), caves (Montano and Henderson, 2013; Cheeptham and Saiz-Jimenez, 2015; Maciejewska et al., 2016), mines (Park et al., 2009; Senhorinho et al., 2018), and marine environments (Rosenfeld and ZoBell, 1947; Burkholder et al., 1966; Patin et al., 2016). The marine environment is a burgeoning source of clinically significant natural products, with over 30,000 previously described and more than 1,000 novel compounds discovered each year since 2008 (Blunt et al., 2016; Lindequist, 2016). Marine biodiversity contains an array of secondary metabolites synthesized by marine microfauna and microflora which are driving the focus of scientific research (Figure 3; Leary et al., 2009). Ocean exploration for new natural products has been driven heavily by medicinal need. However, there is a lack of research on the ecological importance of antimicrobial compounds within natural habitats.

The ecology of microorganisms is complex, due to the number of individuals and vast diversity of natural communities. Fierce competition is driven by a variety of factors such as finite nutrient availability, access to sunlight, and a lack of space. There are two mechanisms of competition. The first is a passive approach, which is resource consumption thus causing indirect competition (Hibbing et al., 2010; Morris, 2015; Ghoul and Mitri, 2016). The second method is through direct interference where microorganisms attempt to “fight” one another through use of chemicals or toxins (Hibbing et al., 2010; Ghoul and Mitri, 2016). Antimicrobial production is the most well-known example of direct competition. Natural products produced by microorganisms are weaponized to gain a competitive advantage for essential nutrients and space (Chao and Levin, 1981; Riley and Gordon, 1999; Abrudan et al., 2015; Cornforth and Foster, 2015).

Antimicrobials can be divided into many chemical classes and can have a range of actions against neighboring cells. Antimicrobials can be broken down into two large groups: β-lactams and non-β-lactams (Donowitz and Mandell, 1988; Clairoux et al., 1992). Within β-lactams the major classes are penicillins, β-lactams, cephems, and penems (Clinical and Institute, 2009). The major classes within non-β-lactams include aminoglycosides, glycopeptides, lipopeptides, macrolides, quinolones, and tetracyclines (Clinical and Institute, 2009). These classes all have various mechanisms of action which include inhibition of cell wall synthesis, protein synthesis, DNA replication and repair, and disruption of the cell membrane (Epand and Vogel, 1999; Lewis, 2013). Antimicrobials also have a range of toxicity which affects the minimum inhibitory concentration (MIC) (Vogelman and Craig, 1986). The MIC is defined as the minimum concentration of an antimicrobial to inhibit growth (Vogelman and Craig, 1986; Li et al., 2017). Antimicrobial MICs can vary between classes and can be affected by surrounding cell concentration and cell type such as resistant, persistent, dormant, biofilm-associated or planktonic microorganisms (Li et al., 2017). Based on the respective environment, antimicrobial toxicity can vary. Secondary metabolites including antimicrobials may serve additional roles in communication and cooperation between and among species.

Antimicrobials may also be utilized as a means of communication, i.e. quorum sensing, or enabling commensal or mutualistic relationships within microbial communities (Ghoul and Mitri, 2016). Low doses of potentially harmful environmental stimuli such as of antimicrobials may produce beneficial effects in microbial cells *via* a process termed hormesis (Southam, 1943; Kendig et al., 2010). The concept of hormesis is not new (Stebbing, 1982); however, antimicrobial-induced hormesis has become a new avenue of research (Yim et al., 2007; Mathieu et al., 2016; Okada and Seyedsayamdost, 2017). Different concentrations of antimicrobial compounds may result in various ecologically significant hormetic effects (Stebbing, 1982; Calabrese, 2005; Davies et al., 2006) influencing the expression of genes potentially involved in elevated virulence in pathogenic Bacteria (Davies, 2006; Linares et al., 2006; Mathieu et al., 2016; Arseneault and Filion, 2017; Dersch et al., 2017) increased biofilm formation (Hoffman et al., 2005; Ranieri et al., 2018) and mutation frequency (Gillespie et al., 2005; Henderson-Begg et al., 2006), stimulation bacterial adhesion (Fitzpatrick et al., 2002), and enhanced gene transfer (Wang et al., 2005). Diverse classes of antimicrobials have been analyzed to determine their effects on bacterial physiology. For instance, beta-lactam antibiotics that inhibit cell wall biosynthesis have been found to increase biofilm formation, while antimicrobials such as fluoroquinolones targeting specific cellular functions like DNA replication may decrease formation (Marti et al., 2017; Ranieri et al., 2018; Yu et al., 2018). Likewise, some antimicrobials can alter bacterial cell surface properties such as ionic charge and cause more favorable conditions for adherence and biofilm formation (Kumar and Ting, 2013; Kumar and Anthony, 2016; Ranieri et al., 2018). The specific roles of antimicrobial compounds in nature are highly debated, which increases the need for further study in natural environments (Davies, 2006; Keren et al., 2013; Dwyer et al., 2015; Ranieri et al., 2018). The prevalence of antimicrobial-driven symbioses has been demonstrated within terrestrial and marine ecosystems (Hardin, 1960; Hutchinson, 1961; Fredrickson and Stephanopoulos, 1981; Smith and Waltman, 1995; Fontaine et al., 2003; Prosser et al., 2007; Coyte et al., 2015), yet the majority of such studies were conducted using traditional culture-dependent techniques under controlled laboratory conditions, and therefore not representative of the natural environment.

Antimicrobial production in the environment is a relatively young area of research (Riley and Gordon, 1999; Woon and Fisher, 2016; Van Der Meij et al., 2017), which appears to result in a wide range of ecological effects. An antimicrobial compound can cause surrounding cells to enter dormancy (Gilbert et al., 1990; Song et al., 2019), inhibition of specific cellular functions such as DNA replication and repair (bacteriostatic) (Vogelman and Craig, 1986; Lewis, 2013), death (bactericidal) (Nastro and Finegold, 1972; Vogelman and Craig, 1986; Peterson and Shanholtzer, 1992; Voo et al., 2016), become tolerant to the secondary metabolite (Lewis, 2017), or become resistant to the antimicrobial (Cohen, 1992; Riley and Gordon, 1999; Woon and Fisher, 2016). Cells that detect a molecule that is not favorable can go into dormancy until more favorable conditions return (Song et al., 2019). Some dormant cells could become persister cells which are highly tolerant to antimicrobial compounds (Lewis, 2007, 2008, 2010). Cells that become tolerant or resistant to antimicrobials will be able to withstand the metabolites and even proliferate while other populations are inhibited (Lewis, 2010, 2017). It is plausible that all of these events could be occurring simultaneously, which could culminate in shifts in microbial diversity thereby altering the prevailing metabolic function of the environment (Woon and Fisher, 2016). The key to understanding such a theory would be to monitor the environment over time and document shifts as a result of antimicrobial production. As such, there is a lack of natural ecological data in regard to what drives different, uncultured, and/or under-studied microbial taxa to produce antimicrobials (Hibbing et al., 2010; Zhu et al., 2014; Van Der Meij et al., 2017).

## Archaeal Antimicrobials

Antimicrobial production in Bacteria has been studied for over 85 years and Eukarya have been studied for almost 60 years, but Archaea have received comparatively little attention (O’connor and Shand, 2002; Shand and Leyva, 2007). Proteinaceous archaeal antimicrobial compounds (i.e., archaeocins) were discovered in halophiles in 1982 (O’connor and Shand, 2002), and were once thought to be limited to extreme halophiles (i.e., halocins) until sulfolobicin was described in *Sulfolobus islandicus*, a hyperthermophilic Crenarchaeote found from solfataric fields in Iceland (Prangishvili et al., 2000; O’connor and Shand, 2002). Halocin production has been described as universal across rod-shaped archaeal halophiles, but only a small portion have been characterized (Shand and Leyva, 2007; Mandal et al., 2014). There have been six categories proposed (Rodriguez-Valera et al., 1982), but the type of halocin produced is dependent on the producing microorganism, the surrounding environmental conditions, and growth phase of the culture (Besse et al., 2015). Halocins cause membrane deformation and inhibits Na^+^/H^+^ antiporter in halobacteria (Demain et al., 2019). Sulfolobicins have been found to belong to only members of *Sulfobales* and affects membrane structure (Demain et al., 2019; Kumar and Tiwari, 2019). Both halocins and sulfolobicins have different structures than bacterial antimicrobials that affect Archaea, which could be indicative of unique compounds yet to be surveyed for medicinal purposes (Nagrale and Gawande, 2018).

Polymorphic toxin systems (PTSs) are used by Bacteria against similar strains or species by cleaving the toxin domain off of the protein upon entry into the neighboring cells (Zhang et al., 2012a; Jamet and Nassif, 2015). The toxins released from the PTSs can attack a wide array of targets including nucleic acids, lipids, and proteins (Zhang et al., 2012a; Makarova et al., 2019). Archaeal homologs of PTSs have been identified but have not been fully explored (Makarova et al., 2019). Makarova et al. surveyed archaean genomes and found 141 genomes to contain genes predicted to encode PTSs (Makarova et al., 2019). Many of the archaeal genomes did not have recognizable self-defense mechanisms compared to bacterial defenses, which can suggest novel or unique self-defense mechanisms that could be creating toxic compounds with antimicrobial activity (Makarova et al., 2019).

The number of cultured Archaea is few compared to Bacteria and fungi; therefore, bioprospecting for antimicrobially active natural products is currently limited in laboratory settings (Gagen et al., 2013; Besse et al., 2015). There has been very little focus on secondary metabolites with potential antimicrobial activity within Archaea (Kumar and Tiwari, 2019; Makarova et al., 2019). Archaea likely contain an array of novel or unique antimicrobial biosynthesis pathways (Roller and Gowan, 2016) that may be elucidated through culture-independent methods, especially considering the widespread distribution of this domain in non-extreme terrestrial and marine environments (Stein and Simon, 1996; Karner et al., 2001; Francis et al., 2005; Biddle et al., 2006; Leininger et al., 2006; Lipp et al., 2008; Zhang et al., 2012b; Seitz et al., 2016; Hoshino and Inagaki, 2019).

## Continental Soils, a Fertile Ground for Antimicrobial Discovery

Continental environments are diverse, but only comprise approximately 30% of Earth’s surface. Soils are dynamic due to their heterogenous nature and microbes there are diverse (Raaijmakers and Mazzola, 2012). Soils experience spatial and temporal variation from both abiotic and biotic factors, which can cause microbes to experience difficulty in proliferating and surviving (Raaijmakers and Mazzola, 2012). Nutrient limitation, e.g., nitrogen and phosphorus, as well as increased cellular abundance have been found to induce microbial competition mechanisms including antimicrobial production (Bibb, 2005; Ghoul and Mitri, 2016; Dundore-Arias et al., 2019). Natural product production has been extensively studied in soils since the discovery of penicillin in 1929 (Ligon, 2004).

Although antimicrobials are phylogenetically widespread throughout Bacteria and portions of Archaea and Eukarya, most studies in continental soils have focused on a relatively small fraction of microorganisms that are genetically predisposed to producing antimicrobial compounds (Fiedler et al., 2005; Tiwari and Gupta, 2012). Some of the most notable lineages include *Actinomycetales* (i.e. actinomycetes), *Myxobacteria*, *Bacillus* and, to a lesser extent, some archaeal lineages, filamentous fungi, and *Cyanobacteria*. The gram-negative *Actinobacteria*, especially *Streptomycetes* spp. and spore-forming actinomycetes, produce bioactive compounds demonstrating antibacterial, antifungal, and antitumor activities (Berdy, 1995; Lazzarini et al., 2000). More than 10,000 known bioactive products have been discovered in *Streptomyces* (Bérdy, 2012; Weber et al., 2015), while some actinomycetes are capable of creating 30–50 secondary metabolites (Katz and Baltz, 2016). Genomic analyses from different ecosystems, such as soils and sediments, have found that between 5 and 10% of actinomycete genes are for secondary metabolite synthesis, including antimicrobial compounds (Nett et al., 2009; Ghoul and Mitri, 2016).

Although there has been considerable focus on actinomycetes and *Streptomycetes*, the capacities of these well-studied genera have yet to be exhausted as new genes are currently being discovered from these organisms. For example, new products with antimicrobial activity such as thiopeptide antibiotics have been isolated from *Streptomyces* (Blunt, 2009; Nandhini et al., 2018; Schneider et al., 2018). Cryptic gene clusters, or biosynthetic clusters that have yet to be identified, have recently been described in this genus *via* culture-independent data mining through genomics (Aigle et al., 2014; Antoraz et al., 2015; Weber et al., 2015). Thus, both novel and known actinomycetes likely produce uncharacterized secondary metabolites. *Myxobacteria* have also been shown to contain novel antimicrobial producing gene clusters but are typically difficult to culture (Strohl, 2004). *Bacillus* spp., (a member of *Firmicutes* phyla) is a prolific producer of cyclic peptides and polyketides that have limited overall structural diversity (Tillett et al., 2000; Stein, 2005). Shifting research to these underexplored groups such as *Firmicutes* can hold more potential for novel or unique antimicrobial production.

There is some debate regarding the further exploration of continental environments for bioactive molecules. Most literature supports prospecting in unexplored and underexplored ecosystems (Indraningrat et al., 2016; Danilovich et al., 2018), while some still support exploring soil for novel bioactive molecules (Daniel, 2004; Borsetto and Wellington, 2017). One explanation for this debate is the complexity of soils, as they contain distinct microhabitats of differing physicochemical gradients and environmental conditions (Torsvik and Øvreås, 2002) that host diverse microorganisms whose specific metabolisms and interactions are integral to biogeochemical cycling (Daniel, 2004). Additionally, continental soils have the largest microbial abundance per gram in comparison to other ecosystems (Killham, 1994), and complex communities with closely co-located populations may support novel members with the potential to produce secondary metabolites (Crits-Christoph et al., 2018). While these interconnected systems and the secondary metabolites sourced from them have been studied for decades, the limitations of culture-based techniques has led to a drastic decrease in the discovery rate of novel secondary metabolites since the Golden Age (Strohl, 2000; Daniel, 2004). This leaves the remaining question: what are we missing? Culture-independent sequencing methods have pointed us in the right direction of potentially undiscovered natural products or unknown derivatives of known bioactive molecules in alternative environments such as freshwater, marine, and deep subsurface systems.

## Freshwater Exploration

Antimicrobial production exploration has extended to freshwater environments over the past few decades (Sibanda et al., 2010; Bharathi et al., 2011; Saravanan et al., 2015; Passari et al., 2017). Much of the research surrounding freshwater antimicrobials has focused on *Cyanobacteria* due to their contributions to carbon cycling (Stanier and Bazine, 1977; Carmichael, 1994; Herrero et al., 2001; Rai, 2018) and production of toxins that act as direct competition mechanisms (Swain et al., 2017). *Cyanobacteria* genomes have been shown to contain many gene clusters of PKSs and NRPSs (Christiansen et al., 2001; Schembri et al., 2001; Moffitt and Neilan, 2003; Ehrenreich et al., 2005; Pancrace et al., 2017; Zhang et al., 2017; Demay et al., 2019; Maurya and Mishra, 2019). There have been at least 33 PKS or NRPS clusters experimentally identified within *Cyanobacteria* and some have been linked to the production of microcystin and anabaenopeptilide in freshwater and marine environments (Pancrace et al., 2017). Microcystins and anabaenopeptilides are secondary metabolites that inhibit protein phosphatases and serine proteases, respectfully (Repka et al., 2004). Both of these toxins can be used to inhibit protein synthesis thus inhibiting surrounding microorganisms from proliferating. Multiple species of *Cyanobacteria* produce antimicrobials that defend against harmful pathogens such as *B. subtilis*, *S. aureus*, *Streptococcus mutans*, and *E. coli* (Madhumathi et al., 2011). *Microcystis aeruginosa* and *Nodularia spumigena* produce microcystin and nodularin, which are used to inhibit surrounding community members (Moffitt and Neilan, 2000, 2001; Tillett et al., 2000). However, analyses of the ketosynthase domain within PKS genes have revealed more biosynthetic diversity within *Cyanobacteria* genera including *Cylindrospermopsis* and *Umezakia* (Moffitt and Neilan, 2003). Freshwater *Cyanobacteria* encode diverse PKSs and NRPSs are and able to produce a wide array of antimicrobial compounds including cyclic peptides, macrolides, and terpenoids (Ehrenreich et al., 2005; Busti et al., 2006; Frangeul et al., 2008; Silva-Stenico et al., 2011; Swain et al., 2017). These studies suggest that *Cyanobacteria* may contain uncharacterized biosynthetic gene clusters or create novel compounds. Additionally, it has been suggested that undifferentiated filamentous and heterocystous strains (e.g., *Anabaena*, *Nodularia*, *Nostoc*, and *Spriulina*) show the greatest potential for natural product biosynthesis (Ehrenreich et al., 2005). Over 60% of *Cyanobacteria* genera are capable of biosynthesizing secondary metabolites with putative antimicrobial activity (Demay et al., 2019). There have been 260 compound families of *Cyanobacteria* secondary metabolite products and can have a range of activities including lethality, cytotoxicity, antibacterial, anti-microalgal, and enzyme inhibition (Demay et al., 2019). Due to their PKS and NRPS diversity (Brito et al., 2015; Mazard et al., 2016), *Cyanobacteria* will likely be the primary focus for future exploration of novel or unique antimicrobials in freshwater environments. Putative antimicrobial activity is suspected based on the discovered gene clusters; however, many of the gene clusters have only been observed from a culture-independent standpoint (Demay et al., 2019). *Cyanobacteria* also have been heavily investigated for the toxin production that can have an antimicrobial affect (Carmichael, 1992, 1994; D’agostino et al., 2016). Detection of PKS and NRPS domains is an important first step in identifying putative antimicrobial production. To definitively state that antimicrobial production is occurring, both culture-dependent and culture-independent needs to be performed. Determining the ecology of *Cyanobacteria* secondary metabolite producers would require culture-dependent methods to have concrete evidence of antimicrobial activity (Demay et al., 2019).

Many Bacteria isolated from freshwater environments have shown antimicrobial activity including *Actinobacteria* and *Proteobacteria* which are typically highly abundant phyla in freshwater sediment communities (Zhang et al., 2015; Zothanpuia et al., 2016; Chung et al., 2018; Nam et al., 2018; Passari et al., 2018; Koch et al., 2019). Isolates collected from sediments in Tamdil Lake, India belonged to 10 different genera and seven of the isolates had type II PKS and NRPS (Zothanpuia et al., 2016; Passari et al., 2018). Detection of these genes was performed using PCR selecting for the ketosynthase domains and adenylation domains for PKS and NRPS, respectively (Zothanpuia et al., 2016). Along with the detection of PKS and NRPS domains, the isolates were grown and tested for antimicrobial production by analyzing the zones of clearing, also known as zones of inhibition, around the inoculation site (Zothanpuia et al., 2016). The isolates containing biosynthetic gene domains were identified as *Staphylococcus, Methylobacterium, Lysinibacillus, Bosea, Aneurinibacillus, Bacillus*, and *Novosphingobium* (Zothanpuia et al., 2016). Detection of biosynthetic gene clusters in these isolates is the first step in determining the ecology of PKS and NRPS metabolites *in situ*. Determining what metabolite is created, the mechanism of action can be determined which can then elucidate what that metabolite is being used for against surrounding community members. All of these genera have been underexplored for antimicrobial production, especially *Novosphingobium*, which could indicate an untapped reservoir for unique or novel antimicrobial genes (Zothanpuia et al., 2016). An isolate from Nakdong River, South Korea was identified as *Paucibacter aquatile* and contains nine biosynthetic gene clusters that are involved in lantipeptide, bacteriocin, terpene, and NRPS biosynthesis (Chung et al., 2018). Lantipeptides and terpenes do extensive damage to microbial cell walls thus killing surrounding cells (Chatterjee et al., 2005; Sieniawska et al., 2017). Lantipeptides and terpenes could be used to lyse surrounding cells to gain access to nutrients harbored by the surrounding cells, eliminate competition, or gain access to spatial resources (Chatterjee et al., 2005; Sieniawska et al., 2017). Bacteriocins are much more diverse in mechanisms of action so these ribosomal produced molecules could inhibit or kill surrounding cells (Yang et al., 2014). Bacteriocins could be used as a competitive advantage to eliminate different types of surrounding microbial cells based on the mechanism of action (Yang et al., 2014). To date, there are only three cultured representatives within *Paucibacter*, which could be a prolific antimicrobial producer based on preliminary screenings and genome analyses (Pheng et al., 2017; Chung et al., 2018; Nam et al., 2018).

## Mining for Antimicrobials

Terrestrial mines have been heavily investigated for microbial communities adapted for the extreme conditions, such as pH, temperature, lack of sunlight, and nutrient limitation, associated with mining activity (Rowe et al., 2007) and acid mine drainage (Baker and Banfield, 2003). Many factors can affect antimicrobial production including oxygen, temperature, pH, carbon, nitrogen, and phosphorus availability (Raaijmakers et al., 2002). Fermentation is the dominant metabolism that creates antimicrobial products (Harms et al., 2017). If there is high oxygen concentration, then fermentation will not occur, thus causing inhibition of antimicrobial production. Microorganisms have optimum temperature and pH for growth and for metabolic activity (Nastro et al., 2011; Wang et al., 2011; Vijayakumari et al., 2013). Temperature can affect the microorganism’s ability to proliferate and metabolize efficiently (Gillooly et al., 2001). The enzymes produced from metabolic activity including products of PKSs and NRPSs can have reduced affinity for its respective substrates if the pH is too far from the microbe’s optimum, which can then cause enzymes to be less efficient (Dixon, 1953). Antimicrobial production can be upregulated in environments with limited phosphate and nitrate availability (Bibb, 2005; Van Wezel and Mcdowall, 2011; Liu et al., 2013; Van Der Heul et al., 2018). Some mines have been characterized to be nutrient limited especially in phosphate such as Sanford Underground Research Facility (Osburn et al., 2014). Mining also produces acid mine drainage which causes a large amount of metals and sulfides to be introduced to the environment (Baker and Banfield, 2003). Metal precipitates in acid mine drainage has been found to have an indirect effect on nutrient availability including phosphorus (Hogsden and Harding, 2011; Denicola and Lellock, 2015). Further examination of mines including the abiotic factors could give insight into regulation of antimicrobial production.

A study conducted on an abandoned coal mine in South Korea found eight antimicrobial compounds from a *Streptomycete* isolate exhibited moderate activity against *M. luteus*, *E. hirae*, and methicillin resistant *Staphylococcus aureus* (MRSA) (Park et al., 2014). Alkaline mines have also been found to host microorganisms capable of antimicrobial production (Hill et al., 2017). A *Nocardiopsis* sp. isolated from alkaline mine waste from China produced Napthospironone A, which exhibited moderate activity against *B. subitilis*, *E. coli, S. aureus*, and *Aspergillus niger* (Ding et al., 2010). This finding suggests that mine-sourced antimicrobials may be used to combat emerging resistant strains of Bacteria such as *S. aureus* and *E. coli*. Further determination of what secondary metabolites are produced from these isolates would give tremendous insight into how the antimicrobial(s) affect the ecology of the surrounding environment. For example if the antimicrobial compounds are determined to inhibit surrounding microbial cells, then we could hypothesize that the antimicrobial producer is trying to gain access to surrounding nutrients or spatial resources. If the antimicrobials lyse surrounding cells, then we could hypothesize a predatory interaction where the producer is using the lysed cell for energy rather than what is available in the surrounding environment.

Many mines are rich in heavy metals including copper, iron, nickel, cadmium, and chromium, which have been found to increase or decrease secondary metabolism in many prokaryotes and fungi (Chakrabarty and Roy, 1964; Weinberg, 1990; Haferburg et al., 2007). *Streptomyces galbus* can produce antifungals when their medium includes copper, zinc, or iron, but can experience reduction of antimicrobial production in the presence of cadmium (Paul and Banerjee, 1983; Raytapadar et al., 1995; Haferburg et al., 2007). Chromium has been used to increase actinorhodin production from *Streptomyces coelicolor* (Abbas and Edwards, 1990; Haferburg et al., 2007). Antimicrobial compounds that are found in the presence of metals can form strong complexes such as chloramphenicol in the presence of calcium, iron, palladium, and gold, which can further enhance their activity against surrounding microorganisms such as *S. aureus*, *E. coli*, *B. subtilis*, and *P. aeruginosa* (Al-Khodir and Refat, 2016). Such complex interactions between microorganisms and the surrounding geochemistry could drive the production of novel compounds not seen in model laboratory systems (Jose and Jebakumar, 2014; Hill et al., 2017). Antimicrobial gene regulation has been studied in the model genus *Streptomyces* and found in nutrient limitation such as nitrate; antimicrobial production is increased (Chakraburtty and Bibb, 1997; Song et al., 2004; Bibb, 2005). Many environments like mines are oligotrophic and are limited in nitrate, which could therefore induce antimicrobial production. This hypothesis of oligotrophy driving antimicrobial production can be used to target specific environments for putative natural antimicrobial production.

## Caving for Unknown Antimicrobials

It is estimated that only 10% of all caves have been discovered and only half of the known caves have been explored (Ghosh et al., 2017). Caves contain isolated environments within Earth’s subsurface that are devoid of sunlight, making them an extreme environment for life (Northup and Diana, 2001). Low nutrients can encourage competition for limited resources which can lead to antimicrobial production to inhibit or eliminate surrounding microorganisms (Kuenen and Bos, 1989; Summers Engel et al., 2001; Fontaine et al., 2003; Zeppilli et al., 2018).

The production of novel antimicrobial compounds has been investigated in continental and marine caves over the past 30 years (Ghosh et al., 2017). Analysis of microbial community structure from various caves has revealed new species of actinomycetes (Groth et al., 1999; Lee et al., 2000; Jurado et al., 2005), which may be promising leads to new antimicrobial products (Ningthoujam et al., 2009; Cheeptham and Saiz-Jimenez, 2015). For example, cave soils from Phatup Cave Forest Park and Phanangkhoi Cave in Thailand yielded four actinomycete-sourced natural product isolates that exhibited inhibition of virulent Bacteria and tumor cells including *Bacillus cereus*, MRSA, and breast cancer (Nakaew et al., 2009). Over 400 actinomycetes isolates from soils and rocks located in Helmcken Falls Cave in British Columbia were screened for antimicrobial activity against *Candida albicans*, *Micrococcus luteus*, *Mycobacterium smegmatis*, *Psuedomonas aeruginosa*, *Acinetobacter baumannii*, *Klebsiella pnuemoniae*, and extended spectrum β-lactamase *Escherichia coli* (Cheeptham, 2013). Many Bacteria have become resistant to existing antimicrobials *via* interaction with surrounding secondary metabolites naturally created and from medicinal purposes thus increasing the need for novel or unique compounds to combat them (Nakaew et al., 2009; Yücel and Yamaç, 2010; Rajput et al., 2012; Cheeptham, 2013; Montano and Henderson, 2013). Future analyses should focus on what types of antimicrobials are produced in order to determine the target for of the secondary metabolites, e.g., cell wall synthesis inhibition or protein synthesis inhibition. Upon discovery of the target, we may be able to determine what is happening *in situ* between the producer and the target.

*Streptomyces* and *Nocardia* isolates from Siberian carbonate cave deposits produced antimicrobial and antifungal compounds (Axenov-Gibanov et al., 2016). These *Streptomyces* are one of the most prolific producers of antimicrobial compounds, and finding isolates that produce antimicrobial compounds within oligotrophic systems further supports the hypothesis of nutrient limitation driving natural product biosynthesis (Maciejewska et al., 2017). Samples from Mystery Cave and Norman’s Cave in Exuma Cays, Bahamas were shown to contain four genera with genes specific for PKS and/or NRPS pathways (Hodges et al., 2012). Submarine caves pose a unique extreme environment for microbial communities including salinity gradients (Fichez, 1990; Sket, 1996), limited nutrients (Ghosh et al., 2017), and anoxic conditions (Jaume and Boxshall, 2009). Thus far few studies have been conducted on microbial ecology in submarine caves that focus on antimicrobial production. Cave exploration for antimicrobial production can lead to more insight into microbial ecology in extreme environments and possibly novel or unique compounds (Zeppilli et al., 2018).

## Diving Into the Abyss for Antimicrobial Discovery

The diversity of oceanic habitats is extensive, ranging from shallow, sunlit neritic zones to the eternal darkness of the hadalpelagic, from eutrophic coastal environments to oligotrophic gyres. Exploiting various environments for antimicrobial compounds have diversified in the past decades to include marine ecosystems (Figure 3), yet many of these have yet to be explored for unique antimicrobial products. Bioinformatic data mining has been used to analyze existing metagenomic datasets to identify putative essential PKS and NRPS domains such as those from the 2006–2007 Galathea 3 global multidisciplinary research expedition (Machado et al., 2015; Marchado, 2016; Kealey et al., 2017). Within this data mining venture, a total of 21 genomes belonging to *Alphaproteobacteria* and *Gammaproteobacteria* were found to contain numerous clusters with potential antimicrobial activity (Machado et al., 2015). Another study that included metagenomic sequencing from the Rabigh coast of Saudi Arabia recovered many taxa (e.g., *Proteobacteria*, *Bacteroides*, *Actinobacteria*, *Cyanobacteria*, *Acidobacteria*, and *Firmicutes*) that contained antibiotic portions of PKS and NRPS gene domains (Al-Amoudi et al., 2016). This study found taxa that are not traditionally targeted for antimicrobial production, like actinomycetes.

*Actinomycetales* have received extensive research in oceanic environments (Zhu et al., 2014; Weber et al., 2015). Actinomycetes can survive a wide range of environmental conditions such as high pressure (maximum of 1,100 atm) (Colquhoun et al., 1998a,b; Kim et al., 2006), temperatures (0–100°C) (Fergus, 1964; Cross, 1968; Whyte et al., 1999; Kim et al., 2006), oxic or anoxic conditions (Goodfellow and Williams, 1983), and pH (2.8–10.5) (Williams et al., 1971; Schippers et al., 2002; Kim et al., 2006; Mehta et al., 2006). The ubiquity of actinomycetes has led to increasing efforts in their recovery and isolation from marine environments for bioprospecting potential antimicrobial compounds. Imada et al. (2007) showed that the removal of seawater from culture media ceased antimicrobial production in marine actinomycetes. This finding supports the idea of certain conditions within a marine ecosystem upregulate the expression of antimicrobial production genes.

The marine environment is highly diverse and contains many dynamic habitats that contain specialized microbial communities. All environmental parameters must be considered when interpreting microorganisms’ function within a specific environment. The nutrients that are available for use can dictate which microbes are present and actively functioning. Physical parameters can affect the biogeography of microorganisms. The marine environment hosts many dynamic systems, which can host diverse microbial communities. A comprehensive approach of analysis including biotic and abiotic data will give more clarity to what is driving microorganisms to produce antimicrobial compounds.

## Drilling Down to Discover Antimicrobials in the Deep Subsurface

The deep subsurface biosphere was generally defined as life present greater than 1 m below seafloor (mbsf) (Jørgensen and Boetius, 2007; Orsi et al., 2013b), but has been updated to establish an age and depositional setting (Kirkpatrick et al., 2016). Subsurface microbial life is widespread, with communities documented in oceanic crust and sediments as deep as 2,458 mbsf (Inagaki et al., 2015). The deep subsurface is considered an extreme environment due to low and high temperatures, high pressure, lack of nutrient/carbon availability, and represents an ideal location to understand how microbial populations co-exist under resource-limited conditions. As depth increases, metabolism slows and growth rate decreases, suggesting that cell maintenance may be the most important factor for survival (Jørgensen and Marshall, 2016). How microbial communities survive (and possibly thrive) within this environment is an ongoing question that has continued to drive research there. To date, research in these environments has predominantly focused on prokaryote metabolism and biogeochemical cycling (Orcutt et al., 2013; Orsi et al., 2013b; Baker et al., 2015; Long et al., 2016; Zinke et al., 2017; Orsi, 2018; Reese et al., 2018). However, no studies to date have focused on the prevalence of antimicrobial production or antimicrobial resistance in the deep subsurface biosphere.

Some studies have identified the potential for antimicrobial production *via* metatranscriptomics (Orsi et al., 2013b; Zinke et al., 2017) as well as culture-dependent techniques (Pathom-Aree et al., 2006). In the latter study, actinomycetes were found from the Mariana Trench and identified using species-specific primers targeting this specific taxon of microorganisms. Of the 38 samples, over half had NRPS gene sequences and nine had PKS Type I gene sequences (Pathom-Aree et al., 2006). Some of the recovered gene sequences encoding for putative secondary metabolite production could not be identified when compared to existing databases. The presence of potentially novel NRPS and PKS genes warrants further research to determine which secondary metabolites are likely produced in the deep subsurface.

Deeply buried sediments from the Peru Margin (Ocean Drilling Program Leg 201, Site 1229D) were used for metatranscriptome analyses to examine putatively active microorganisms functioning within the sediment. This study concluded that *Actinobacteria* were one of the dominant metabolically active groups, as they were represented in every mRNA sample from site 1229D (Orsi et al., 2013a). Secondary metabolite biosynthesis was also found in every depth sampled; however, *Actinobacteria* were not the only taxa producing these PKSs and NRPSs (Orsi et al., 2013b). Other notable taxa producing PKSs and NRPSs include *Bacteroidetes*, *Firmicutes*, and *Proteobacteria* (Orsi et al., 2013b). *Actinobacteria* with PKSs and NRPSs were dominant in shallower samples, but decreased in abundance with increasing depth. The deeper sediment samples contained mostly *Betaproteobacteria* and *Gammaproteobacteria* with PKSs and NRPSs. This finding indicates that there are other taxa producing compounds that could be novel or unique.

**Figure 4:**
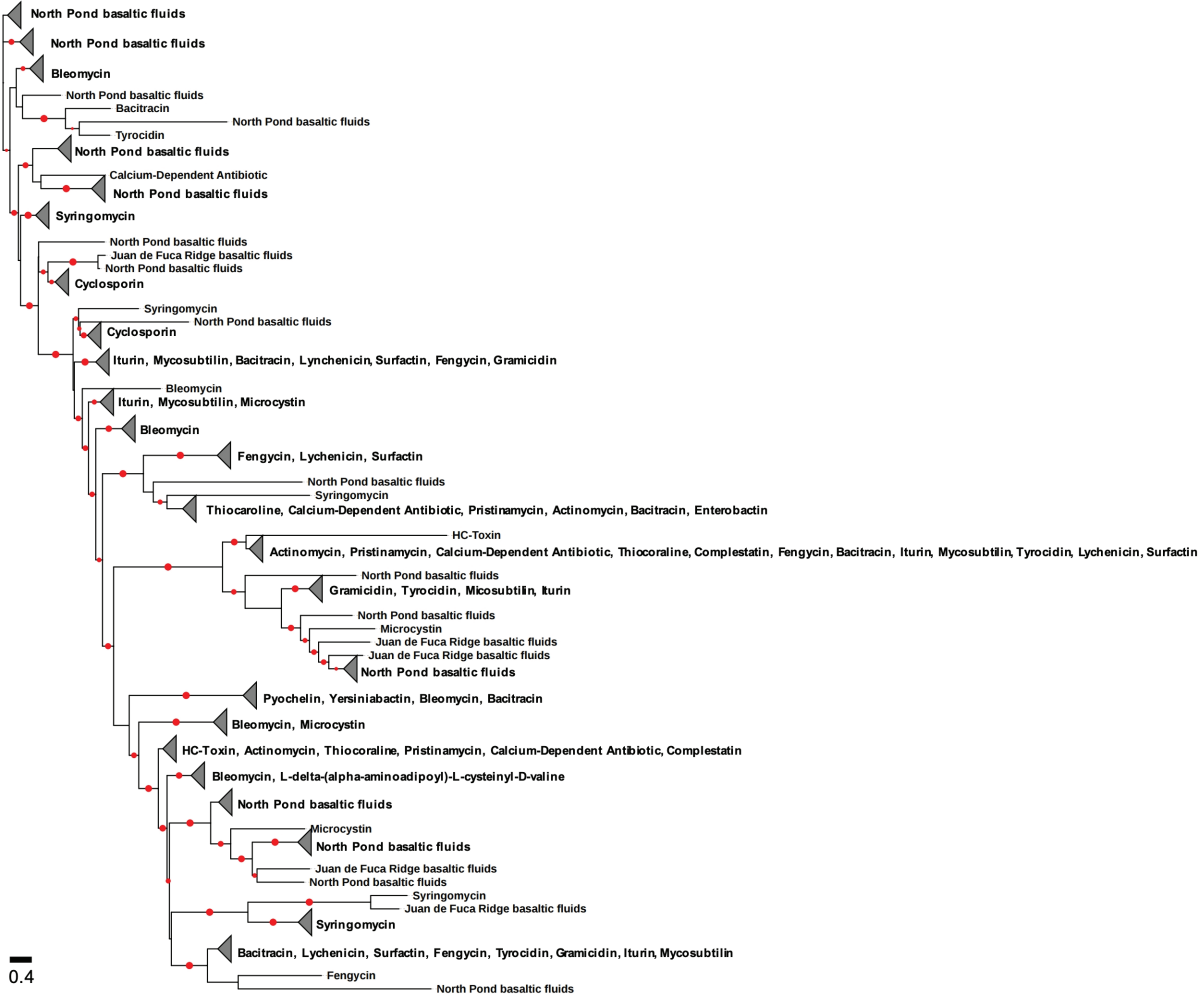
Phylogenetic tree of the condensation domain of the NRPS gene including sequences from terrestrial deep subsurface and marine deep subsurface sites. The reference sequences are from the Natural Product Domain Seeker (NaPDoS) repository. The alignment and tree were built using the NaPDoS pipeline (Ziemert et al., 2012). Red dots indicate bootstrap values greater than 50%.

**Figure 5:**
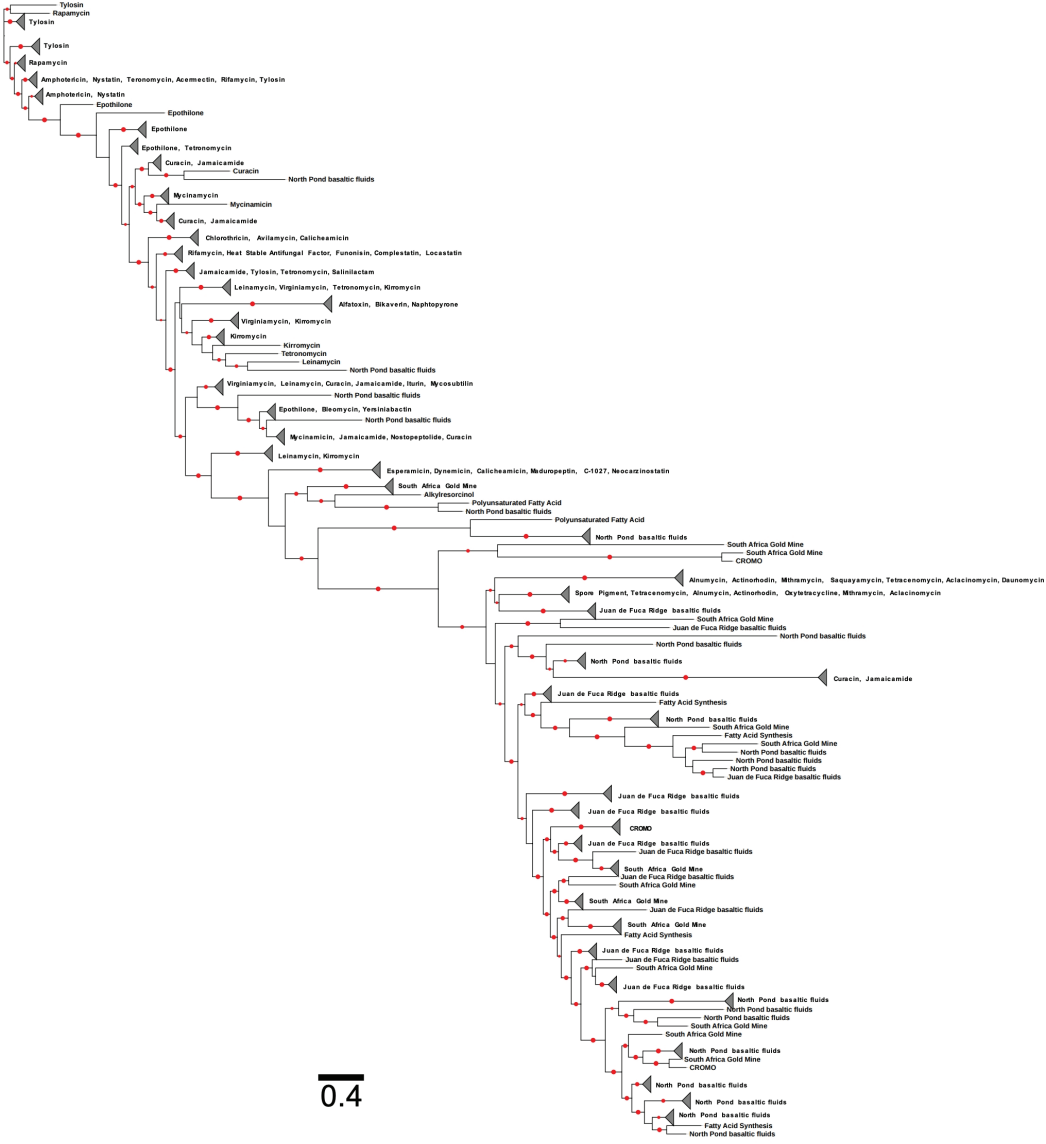
Phylogenetic tree of the ketosynthase domain of the PKS gene including sequences from terrestrial and marine deep subsurface sites. The reference sequences are from the Natural Product Domain Seeker (NaPDoS) repository. The alignment and tree were built using the NaPDoS pipeline (Ziemert et al., 2012). Red dots indicate bootstrap values greater than 50%.

Assuming *Actinobacteria* to be the most prolific producers of antimicrobials, the discovery and isolation of novel members of these lineages provide promise for antimicrobial activity in other subsurface environments (Thornburg et al., 2010). Actinomycete species have been found from deep-sea environments including hydrothermal vent fluids within the Mariana Trough and Suiyo Seamount (Kysela et al., 2005) and hydrothermal sediments of Guaymas Basin (Naganuma et al., 2007). Many deep subsurface environments have been explored through ‘omics’ to analyze microbial community structure and function, but in doing so have also stumbled upon a hotbed of actinomycete lineages. Bacterial 16S rRNA gene sequences related to actinomycetes have been found in deep ocean water column and sediment (Cole et al., 2007, 2008; Huber et al., 2007; Thornburg et al., 2010). In the course of this review, we used assembled metagenomic data collected from IODP Expedition 327 to Juan de Fuca Ridge basaltic fluids (Jungbluth et al., 2017), the IODP Expedition 336 to the western Flank of the Mid-Atlantic Ridge basaltic fluids (Meyer et al., 2016), South Africa Gold Mine (Lau et al., 2014; Magnabosco et al., 2014), and the Coast Range Ophiolite Microbial Observatory (Twing et al., 2017) and analyzed for putative antimicrobial production using the NaPDoS pipeline (Ziemert et al., 2012). The western Flank of the Mid-Atlantic Ridge is commonly referred to North Pond due to the large pond of sediment located on the western side of the ridge above basalts (Langseth et al., 1992). The condensation domain of the NRPS gene was found in all deep subsurface data analyzed through NaPDoS (Figure 4), illustrating the diversity of antimicrobial-related sequences within deeply buried marine environments. In contrast, the ketosynthase domain of the PKS gene was found in all subsurface sites for fatty acid synthesis (Figure 5). Only samples collected from North Pond basaltic fluids contained ketosynthase-related sequences associated with antimicrobial production. Fatty acid synthesis and antibiotic production genes are very similar to one another, and antimicrobial PKSs may be derived from fatty acid synthesis genes (Hopwood, 1999; Metz et al., 2001; Tsai, 2018). The similarity between antimicrobial PKSs and fatty acid synthesis genes may explain why there are two distinct clades (Figure 5). Many marine Bacteria have expressed fatty acid synthesis genes with homology to PKS genes (Metz et al., 2001), particularly within subsurface environments. Sedimentary (and basalt-hosted) microorganisms could be utilizing the PKS gene to synthesize fatty acids necessary for cellular structures like the cell membrane (Metz et al., 2001; Selvin et al., 2016; Tsai, 2018). The deeply buried marine biosphere may host novel or unique antimicrobial activity that contributes to the intricate ecology of the system, microbe-microbe interactions, and habitability.

Here, we illustrate the usefulness in metagenomic studies to elucidate putative antimicrobial production as well as the caveats of such an approach. The possibility of capturing antimicrobial producing genes from solely culture-independent studies can save time and resources (Handelsman et al., 1998; Banik and Brady, 2010; Garcia et al., 2018). Metagenomic analyses are valuable in that researchers can identify putative metabolic processes including antimicrobial production (Bergmann et al., 2007). Although, DNA-based analyses is an excellent first step at identifying potential microbial activities, metagenomic analyses can also detect dormant, dead, or low metabolically active community members (Davis et al., 1986; Orcutt et al., 2013). Taken as a hypothesis-driving guide, metagenomics can uncover hidden potential for antimicrobials, but ideally metatranscriptomics, proteomics, and metabolomics should be performed. A combination of approaches to analyze antimicrobial production would be more efficient and supportive. Thus far many studies have only surveyed antimicrobial production *via* gene-based (Moffitt and Neilan, 2003; Zothanpuia et al., 2016; Demay et al., 2019), culture-dependent (Nakaew et al., 2009; Ding et al., 2010; Cheeptham, 2013; Axenov-Gibanov et al., 2016), and metagenomic studies (Machado et al., 2015; Al-Amoudi et al., 2016; Kealey et al., 2017). Most surveying for antimicrobial production has previously focused on detection of PKS and/or NRPS domains by PCR amplification (Moffitt and Neilan, 2003; Ehrenreich et al., 2005; Pathom-Aree et al., 2006; Hodges et al., 2012; Zothanpuia et al., 2016; Passari et al., 2018). This has been an enlightening approach to survey for putative antimicrobial production. However, this process only identifies one domain present within a given sample. An ‘omics’ approach will give the opportunity to find longer portions and possibly the entirety of PKS and NRPS genes. Metagenomics has become a more prominent source for surveying natural environments but is limited in determining activity. Metatranscriptomics, proteomics, and metabolomics would yield more concrete results of antimicrobial production (Orcutt et al., 2013).

## Putting Antimicrobials on the Map

There is a pressing need for increased exploration of antimicrobials in natural environments due to the lack of information regarding the ecological roles of these compounds. The most favorable locales for bioprospecting can be determined through microbial biogeography, which encompasses spatial distributions, dispersal, and inter- and intraspecies interactions (Martiny et al., 2006) to better understand microbial communities across various environments (Coleman et al., 2006). Analysis of environmental and geochemical parameters including temperature, salinity, pH, depth, nitrogen, carbon, and phosphorus can be used to gain insight to why these microorganisms are producing antimicrobial compounds. This data collection strategy can be applied to each environment sampled for biosynthesis exploration. Each environment hosts a unique ecosystem that shapes how microbial communities interact and survive. A biogeographical approach not only incorporates the traditional methods of isolating bioactive compounds from natural environments, but also includes bioinformatic methods that aid in the discovery of uncultured microbes with the genetic potential to produce novel or unique antimicrobials. Assessing spatiotemporal trends in antimicrobial production through biogeography would provide a means to predict the presence and prevalence of antimicrobial compounds in different ecosystems. Yet, the few published studies on the biogeography of antimicrobial production have shown that spatial distributions are directly correlated with sequence differences in antimicrobial genes (Reddy et al., 2014; Morlon, 2015; Charlop-Powers et al., 2015). PKS and NRPS genes from environments in close proximity were more similar than those that were globally distant (Charlop-Powers et al., 2015), which is to be expected.

Future antimicrobial-focused biogeography studies may benefit greatly from the vast quantities of publicly-available sequencing data, such as the creation and curation of maps of sample isolation sites within the eSNaPD (Reddy et al., 2014) which represent hot spots for antimicrobial production. While these tools are useful in searching for antimicrobial producing genes, it must be considered that data mining approaches are only good as the databases provided (Medema et al., 2011; Ziemert et al., 2012; Reddy et al., 2014). Creating a hand-curated database containing each essential domain that could be present within the PKS or NRPS gene would be more useful to deduce antimicrobial production potential. Existing databases are a good resource for data mining; however, the results will be heavily dependent on the database. Constructing a comprehensive database that includes all variants of genes of interest, such as antimicrobial production, will give the best chance in correctly identifying putative functional genes (Boolchandani et al., 2019). Using one essential domain offers preliminary analysis of presence-absence; however, using multiple domain searches is ideal for supporting antimicrobial production from only culture-independent data. Using a multi-domain search approach for antimicrobial production will give more concrete evidence of putative antimicrobial production. Based on the domains present and the organization of the domains will be more efficient in determining functionality of the secondary metabolite. A single domain offers support of putative antimicrobial production because causes difficulty in determining the secondary metabolite that it encodes. Constructing a large-scale biogeography that encompasses various environments accompanied with physical and geochemical data can give tremendous insight into future exploration. A streamlined guide to antimicrobial exploration will offer better chances to understand microbial ecology and potentially discover novel compounds.

## Future Directions

Generally, the microbial bioactive compounds that have been discovered come from actinomycetes (45%), fungi (38%), and unicellular eubacteria (17%) (Mahajan and Balachandran, 2012), but these numbers do not reflect the potential discoveries that remain if we expand our studies to include underexplored ecosystems and additional domains of life. The studies reviewed here have provided a valuable foundation for antimicrobial exploration and bioprospecting by demonstrating the presence/absence of the PKS and NRPS genes. However, we know that for secondary metabolite biosynthesis to occur, PKS and NRPS antimicrobial genes necessitate every one of their three essential domains, yet research thus far has solely focused on one domain to indicate antimicrobial production. Future studies from all environments must incorporate all three essential domains to effectively demonstrate putative antimicrobial production or risk mischaracterizing the prospect of biosynthesis. This necessitates the need for more complete databases to include all domains. Additionally, the vast majority of studies have utilized only the PKS or NRPS gene (i.e., DNA) as a proxy for antimicrobial activity, which does not necessarily mean that it is being expressed. Using metatranscriptomics as a proxy for microbial activity and gene expression is a first step in overcoming this issue. Lastly, in order to delve into a more thorough understanding of bioprospecting, we must first establish commonalities among sites with high frequency of antimicrobial genes. In order to do this, a myriad of environments must be explored, and abiotic data must be collected. This will provide a more holistic approach to what drives putative antimicrobial production and will give invaluable insight into microbial ecology in natural environments.

Further exploration of antimicrobial production within microbial communities will not only provide a more comprehensive understanding of their ecology with regards to overarching factors such as biogeochemical cycling but also increase potential natural product discovery. Antimicrobial compounds are utilized by specific microorganisms for competition purposes to obtain vital nutrients and spatial resources for survival. Thus far, our knowledge of antimicrobials has been from studies focused on terrestrial environments. However, the marine deep subsurface, one of the largest habitats on Earth, has yet to be explored for antimicrobial genes and products. Understanding the ecology of microbial communities in harsh, remote environments can aid in determining the limits of life as well as microorganisms’ contributions to overall ecology through competition.

## Author Contributions

MM and BR developed the idea for the review. MM and IR analyzed data. MM and BR wrote the review. BB contributed ideas and assisted in editing.

### Conflict of Interest

The authors declare that the research was conducted in the absence of any commercial or financial relationships that could be construed as a potential conflict of interest.
